# Hematological parameters: is there a difference between those released by the hematological analyzer and to the customer?

**DOI:** 10.31744/einstein_journal/2023AO0501

**Published:** 2023-12-01

**Authors:** Jhenifer Monique Pinto, Leilismara Sousa Nogueira, Danyelle Romana Alves Rios

**Affiliations:** 1 Campus Centro Oeste Dona Lindu Universidade Federal de São João del-Rei Divinópolis MG Brazil Campus Centro Oeste Dona Lindu , Universidade Federal de São João del-Rei , Divinópolis , MG , Brazil .; 2 Department of Clinical and Toxicological Analysis Universidade Federal de Alfenas Alfenas MG Brazil Department of Clinical and Toxicological Analysis , Universidade Federal de Alfenas , Alfenas , MG , Brazil .

**Keywords:** Blood cell count, Erythrocyte indices, Reticulocyte, Platelets, Laboratory equipment, Laboratories, Surveys and questionnaires

## Abstract

**Objective:**

This study aimed to compare the hematological parameters released by hematological analyzers with those released in customer reports.

**Methods:**

We conducted a descriptive study in the laboratories of a medium-sized municipality in the state of Minas Gerais registered in the National Register of Health Establishments. Interviews were conducted using a questionnaire to obtain information regarding the parameters released by the analyzers and those available in the customer’s report.

**Results:**

Sixteen laboratories were evaluated, and none of them released all the parameters obtained from the hematological analyzers to customers. The red blood cell distribution width was released in 88% of the laboratories, atypical lymphocytes in 70%, mean platelet volume in 50%, platelet distribution width and platelet count in 20%. No laboratory released information on reticulocytes, fraction of immature reticulocytes and immature granulocytes, nucleated erythrocyte count, immature platelet fraction and reticulocyte hemoglobin, and large platelet rate.

**Conclusion:**

All evaluated clinical analysis laboratories had at least one parameter that was not released in the customer’s report despite being released by the hematological analyzers. The lack of knowledge on the part of professionals about the clinical importance of each parameter of the complete blood count results in a loss in patient assessment, and it is important to include these parameters in the complete blood count report.

## Summary

In Brief

Pinto et al. demonstrated that although most hematological analyzers release additional blood count parameters (platelet volume and reticulocyte indices) that are important in the diagnosis of several diseases, they are not released in the client’s report. Therefore, expanding our knowledge and implementing these parameters in the complete blood count is essential.

## Abstract (key points)

Highlights

Recent automated hematology analyzers provide additional parameters to the complete blood count.

Sixteen laboratories of a medium-sized municipality of Minas Gerais were interviewed.

No laboratory released all parameters obtained from the hematological analyzers to the customer.

Expanding our knowledge and implementing these parameters in complete blood counts is essential

## Graphical abstract

**Figure:**
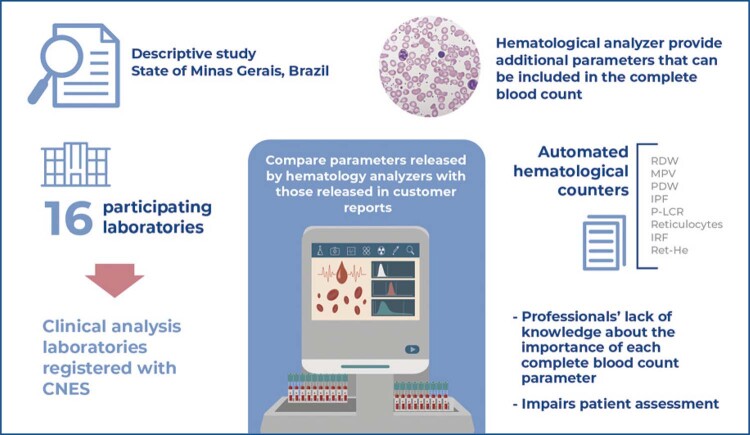

## INTRODUCTION

Complete blood count (CBC) is the most requested test by physicians because it provides important information regarding the production, proliferation, and maturation of different blood cell lineages and is an easily accessible test. ^( 1 , 2 )^ Careful blood analysis is the first step in assessing hematological function and diagnosing related disorders. From the CBC, it is possible to diagnose and morphologically classify anemia, one of the main public health problems in several countries. Moreover, the CBC is indispensable in the diagnosis, prognosis, and monitoring of acute and chronic inflammatory/infectious diseases, hematological neoplasms, and coagulation disorders. ^( 3 )^

In the CBC, the cellular elements present in the blood are evaluated quantitatively and qualitatively. According to the National Quality Control Program (PNCQ). ^( 4 )^ The following parameters must be present in a CBC report: a) erythrogram: number of red blood cells, hemoglobin, hematocrit, and hematimetric indices, such as mean corpuscular volume (MCV), mean corpuscular hemoglobin (MCH), mean corpuscular hemoglobin concentration (MCHC) and red blood cell distribution width (RDW); b) leukogram: global and differential (relative and absolute) leukocyte count; and c) platelet count: number of platelets. ^( 1 , 4 )^ However, although the PNCQ recommends the minimum parameters that must be included in the CBC, the released tests are not standardized.

Automated hematological counters have advanced considerably in recent years, and the latest generation analyzers provide other hematological parameters, which can be added to those already included in the CBC, and which have clinical relevance, such as mean platelet volume (MPV), blood large platelet count (P-LCR), platelet distribution width (PDW), platelet count (PCT), immature platelet fraction (IPF), atypical/abnormal lymphocytes (LIA), immature granulocyte index (IG), nucleated erythrocytes (NRBC), reticulocyte count (RET) and reticulocyte indices such as reticulocyte hemoglobin content (RET-He) and immature reticulocyte fraction (IRF). ^( 5 , 6 )^ The parameters MPV, P-LCR, PDW, PCT, and IPF are known as platelet volume indices (PVIs). ^( 7 )^

These more recent hematological parameters have substantial clinical relevance and enhance the investigation of blood-related diseases, in addition to helping and facilitating a more accurate and reliable diagnosis. However, not all laboratories include them in the CBC report delivered to customers.

## OBJECTIVE

To compare the hematological parameters released by hematological analyzers with those released in a customer report in clinical analysis laboratories in a medium-sized city in the state of Minas Gerais.

## METHODS

###  Study design and location 

This descriptive study was conducted in a medium-sized municipality located in the Midwest region of the state of Minas Gerais, which comprises 56 municipalities. The studied municipality is considered the largest in the region, with an estimated population of 234,937 inhabitants, as a regional health hub, according to IBGE, ^( 8 )^ and has 16 clinical analysis laboratories, four of which belong to hospitals.

### Data collection

Data were collected through a survey of clinical analysis laboratories located in the studied municipality, registered on the National Register of Health Establishments (CNES) website in February 2022. The telephone contacts and addresses of each one of them were also obtained from the website. ^( 9 )^

The second stage of the research was conducted between February 2022 and May 2022 through visits to the laboratories, telephone contact, and institutional emails with those responsible for them. Data were collected through a previously structured questionnaire administered to the laboratory technicians with pertinent and relevant questions for conducting the study (Appendix 1), covering aspects such as the size of the laboratory (small size: up to 20,000 exams; medium size: between 21,000 and 100,000; large size: between 101,000 and 200,000 exams; and extra size: more than 200,000 exams); ^( 10 )^ whether the institution was public or private; the type of care (outpatient, hospital, or both); the device used to perform the CBC test; the method used (flow cytometry, electrical impedance, immunofluorimetry, fluorimetry, photometry, or other); the parameters released by the hematological analyzer, which are validated through the site responsible for its manufacture; and the parameters released in the report that the customer receives (this information was confirmed in the CBC report of each establishment).

After collecting this information, the data were entered into a database (Excel ^®^ spreadsheet) to be evaluated and discussed. It is noteworthy that for privacy reasons, the names of the laboratories in this study were not disclosed. The data used does not involve human beings, which justifies the absence of ethical approval.

### Data analysis

A descriptive analysis of the data was performed to determine the frequencies of the collected information.

## RESULTS

In a search conducted on the CNES website, 22 clinical analysis laboratories were identified in the studied municipality. Of these, one was just a collection point, and five had ceased operation or merged with larger laboratories. Thus, 16 laboratories were included in the study.

Most laboratories were private (n=15, 94%), medium-sized (n=12, 75%), and provided outpatient care exclusively (n=10, 62%).

Table 1 shows the characteristics of the clinical analysis laboratories that were included in the study according to the type of service, institution, and size.

**Table 1:** Characterization of the clinical analysis laboratories participating in the study

Classification	n (%)
Type of service	
Outpatient	10 (62)
Hospital	3 (19)
Outpatient and hospital	3 (19)
Institution	
Private	15 (94)
Public	1 (6)
Size	
Large	1 (6)
Medium	12 (75)
Small	3 (19)

**Figure 1:**
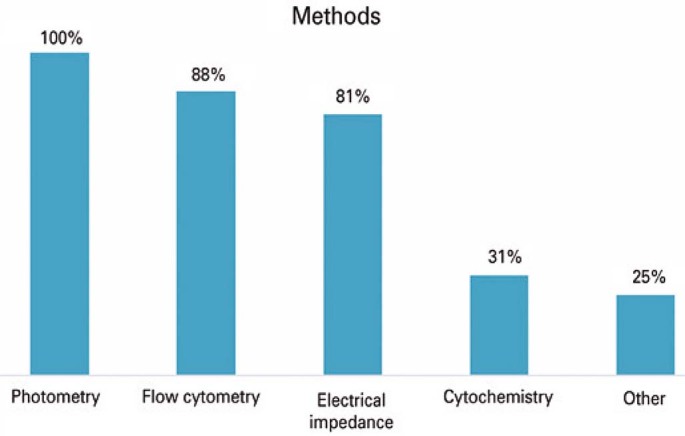
Methods used by hematological analyzers, from the clinical analysis laboratories of the study

Regarding the equipment used, most laboratories use the Horiba ABX Pentra ES 60 hematology analyzer (HORIBA ABX SAS, Kyoto, Japan) (n=3, 19%) and Beckman Coulter DXH 800 (BECKMAN COULTER, Miami/FL, USA) (n=3, 19%), and the most common manufacturer of analyzers used in the municipality was Horiba (n=7, 44%). The frequencies of the methods used in the hematological devices of the evaluated clinical analysis laboratories are shown in figure 1 .

Table 2 presents information regarding the hematological parameters, including the identification of each laboratory (1-16), the parameters released by the hematological analyzers, and those released in the customer’s report.

**Table 2:** Comparison between the parameters released by the hematological analyzer and those released in the reports by the clinical analysis laboratories

Laboratory	Hematology analyzer	Hematology analyzer methods	Parameters released by the hematology analyzer	Parameters released in the customer report
1	CELL-DYN Ruby (Abbott)	Flow cytometry, photometry, MAPSS technology	Hm, Hb, Ht, MCV, MCH, MCHC, RDW (RDW-CV), global leukocyte, neutrophil, lymphocyte, monocyte, eosinophil, basophil, PLT, MPV, PCT, RET	Hm, Hb, Ht, MCV, MCHC, MCHC, RDW, global leukocyte, neutrophil, lymphocyte, monocyte, eosinophil, basophil, PLT, MPV, PCT
2	ABX Pentra ES 60 (Horiba)	Combined DHSS technology, cytochemistry, electrical impedance, photometry, and flow cytometry	Hm, Hb, Ht, MCV, MCH, MCHC, RDW (RDW-CV), global leukocyte, neutrophil, lymphocyte, monocyte, eosinophil, basophil, PLT, MPV, PCT, PDW, LIA, IG	Hm, Hb, Ht, MCV, MCH, MCHC, RDW, global leukocyte, neutrophil, lymphocyte, monocyte, eosinophil, basophil, PLT
3	DXH 800 (Beckman Coulter)	Flow cytometry, electrical impedance, and photometry	Hm, Hb, Ht, MCV, MCH, MCHC, RDW (RDW-SD and RDW-CV), global leukocyte, neutrophil, lymphocyte, monocyte, eosinophil, basophil, PLT, VPM, PDW, LIA, NRBC, RET, IRF	Hm, Hb, Ht, MCV, MCH, MCHC, RDW (RDW-CV), global leukocyte, neutrophil, lymphocyte, monocyte, eosinophil, basophil, PLT, VPM, PDW, LIA
4	ABX Pentra ES 60 (Horiba)	Combined DHSS technology, cytochemistry, electrical impedance, photometry, and flow cytometry	Hm, Hb, Ht, MCV, MCH, MCHC, RDW (RDW-CV), global leukocyte, neutrophil, lymphocyte, monocyte, eosinophil, basophil, PLT, MPV, PCT, PDW, LIA, IG	Hm, Hb, Ht, MCV, MCH, MCHC, RDW, global leukocyte, neutrophil, lymphocyte, monocyte, eosinophil, basophil, PLT, LIA
5	ABX Micros 60 (Horiba)	Electrical impedance and photometry	Hm, Hb, Ht, MCV, MCH, MCHC, RDW (RDW-CV), global leukocyte, neutrophil, lymphocyte, monocyte, eosinophil, basophil, PLT, MPV, PCT, PDW, LIA	Hm, Hb, Ht, MCV, MCH, MCHC, RDW, global leukocyte, neutrophil, lymphocyte, monocyte, eosinophil, basophil, PLT, LIA
6	ABX Pentra XL 80 (Horiba)	Cytochemistry, electrical impedance, flow cytometry, and photometry	Hm, Hb, Ht, MCV, MCH, MCHC, RDW (RDW-CV), global leukocyte, neutrophil, lymphocyte, monocyte, eosinophil, basophil, PLT, MPV, PCT, PDW, LIA, IG	Hm, Hb, Ht, MCV, MCH, MCHC, global leukocyte, neutrophil, lymphocyte, monocyte, eosinophil, basophil, PLT, LIA
7	DXH 800 (Beckman Coulter)	Flow cytometry, electrical impedance, and photometry	Hm, Hb, Ht, MCV, MCH, MCHC, RDW (RDW-SD and RDW-CV), global leukocyte, neutrophil, lymphocyte, monocyte, eosinophil, basophil, PLT, MPV, NRBC, RET, IRF	Hm, Hb, Ht, MCV, MCH, MCHC, RDW (RDW-CV), global leukocyte, neutrophil, lymphocyte, monocyte, eosinophil, basophil, PLT, VPM
8	CELL-DYN Ruby (Abbott)	Flow cytometry, photometry, MAPSS technology	Hm, Hb, Ht, MCV, MCH, MCHC, RDW (RDW-CV), global leukocyte, neutrophil, lymphocyte, monocyte, eosinophil, basophil, PLT, MPV, RET	Hm, Hb, Ht, MCV, MCH, MCHC, RDW, global leukocyte, neutrophil, lymphocyte, monocyte, eosinophil, basophil, PLT
9	ABX Pentra XL 80 (Horiba)	Cytochemistry, electrical impedance, flow cytometry, and photometry	Hm, Hb, Ht, MCV, MCH, MCHC, RDW (RDW-CV), global leukocyte, neutrophil, lymphocyte, monocyte, eosinophil, basophil, PLT, MPV, PCT, PDW, LIA, RET	Hm, Hb, Ht, MCV, MCH, MCHC, RDW, global leukocyte, neutrophil, lymphocyte, monocyte, eosinophil, basophil, PLT, MPV, PCT, PDW, LIA
10	Cell DYN 3700 (Abbott)	Electrical impedance, photometry, and flow cytometry	Hm, Hb, Ht, MCV, MCH, MCHC, RDW (RDW-CV), global leukocyte, neutrophil, lymphocyte, monocyte, eosinophil, basophil, PLT, MPV, PCT, PDW, RET, IRF	Hm, Hb, Ht, MCV, MCH, MCHC, RDW, global leukocyte, neutrophil, lymphocyte, monocyte, eosinophil, basophil, PLT, MPV
11	Yumizen H550 (Horiba)	Flow cytometry, electrical impedance, and photometry	Hm, Hb, Ht, MCV, MCH, MCHC, RDW (RDW-CV and RDW-SD ), leukocyte global, neutrophil, lymphocyte, monocyte, eosinophil, basophil, PLT, MPV, PCT, PDW, P-LCR, LIA	Hm, Hb, Ht, MCV, MCH, MCHC, global leukocyte, neutrophil, lymphocyte, monocyte, eosinophil, basophil, PLT
12	XE-2100 (Sysmex)	Flow cytometry, electrical impedance, and photometry	Hm, Hb, Ht, MCV, MCH, MCHC, RDW (RDW-SD and RDW-CV), global leukocyte, neutrophil, lymphocyte, monocyte, eosinophil, basophil, PLT, IPF, RET, RET-He, IRF, NRBC, IG, LIA	Hm, Hb, Ht, MCV, MCH, MCHC, RDW (RDW-CV), global leukocyte, neutrophil, lymphocyte, monocyte, eosinophil, basophil, PLT, LIA
13	Mindray BC-5000 (Starlab)	Flow cytometry and photometry	Hm, Hb, Ht, MCV, MCH, MCHC, RDW (RDW-CV), global leukocyte, neutrophil, lymphocyte, monocyte, eosinophil, basophil, PLT, MPV, PDW, PCT	Hm, Hb, Ht, MCV, MCH, MCHC, RDW, global leukocyte, neutrophil, lymphocyte, monocyte, eosinophil, basophil, PLT
14	ABX Pentra ES 60 (Horiba)	Combined DHSS technology, cytochemistry, electrical impedance, photometry, and flow cytometry	Hm, Hb, Ht, MCV, MCH, MCHC, RDW (RDW-CV), global leukocyte, neutrophil, lymphocyte, monocyte, eosinophil, basophil, PLT, MPV, PDW, PCT, IG, LIA	Hm, Hb, Ht, MCV, MCH, MCHC, RDW, global leukocyte, neutrophil, lymphocyte, monocyte, eosinophil, basophil, PLT, MPV
15	Coulter LH 750 (Beckman Coulter)	Electrical impedance and photometry	Hm, Hb, Ht, MCV, MCH, MCHC, RDW (RDW-SD and RDW-CV), global leukocyte, neutrophil, lymphocyte, monocyte, eosinophil, basophil, PLT, IPF, RET, RET-He, IRF, NRBC, IG, LIA	Hm, Hb, Ht, MCV, MCH, MCHC, RDW (RDW-CV), global leukocyte, neutrophil, lymphocyte, monocyte, eosinophil, basophil, PLT, LIA
16	DXH 800 (Beckman Coulter)	Flow cytometry, electrical impedance, and photometry	Hm, Hb, Ht, MCV, MCH, MCHC, RDW (RDW-SD and RDW-CV), global leukocyte, neutrophil, lymphocyte, monocyte, eosinophil, basophil, PLT, MPV, NRBC, RET, IRF	Hm, Hb, Ht, MCV, MCH, MCHC, RDW (RDW-CV), global leukocyte, neutrophil, lymphocyte, monocyte, eosinophil, basophil, PLT, MPV

Hm: erythrocyte; Hb: hemoglobin; Ht: hematocrit; MCV: mean corpuscular volume; MCH: mean corpuscular hemoglobin; MCHC: mean corpuscular hemoglobin concentration; RDW: erythrocyte size variability; RDW-SD: determination of anisocytosis; RDW-CV: coefficient of variation of red blood cell distribution amplitude; PLT: platelet count; PCT: platelet count ; PDW: platelet distribution width; MPV: mean platelet volume; P-LCR: large platelet rate; LIA: atypical lymphocytes; IG: immature granulocyte index; IPF: fraction of immature platelets; IRF: fraction of immature reticulocytes; RET: reticulocytes; RET-He: reticulocyte hemoglobin content; NRBC: nucleated erythrocyte count. Parameters highlighted in yellow: parameters that the hematological analyzers perform and that are not released in the blood count report.

Platelet volume was the most common parameter obtained from hematology analyzers, which was not released to customers. Of the 14 (88%) analyzers that released MPV, only 7 (50%) laboratories released it to customers, and 2 (20%) of 10 laboratories released PCT and PDW. Additionally, the P-LCR and IPF indices were released by the hematological analyzers of one (6%) and two (12.5%) laboratories, respectively, and none of them were included in the report.

The RET, IRF, RET-He, and NRBC reticulocyte indices were obtained using the hematological analyzers of nine (56%), six (37.5%), two (12.5%), and five (31%) laboratories, respectively; however, none of them released these results.

Regarding the RDW parameter, all hematological analyzers in this study provided this index; however, two laboratories (12.5%) did not release it in their customer reports. In addition, six (37.5%) analyzers released RDW-CV and RDW-SD; however, no laboratory reported RDW-SD in the customer report.

**Figure 2:**
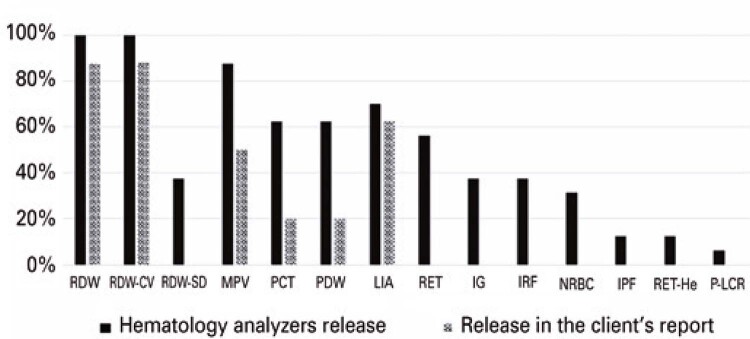
Percentage of the comparison between the parameters released by the hematological analyzers with those released in the client’s report

RDW: erythrocyte size variability; RDW-SD: determination of anisocytosis; RDW-CV: coefficient of variation of red blood cell distribution amplitude; MPV: mean platelet volume; PCT: platelet count; PDW: platelet distribution width; LIA: atypical lymphocytes; RET: reticulocytes; IG: immature granulocyte index; IRF: fraction of immature reticulocytes; NRBC: nucleated erythrocyte count; IPF: fraction of immature platelets; RET-He: reticulocyte hemoglobin content; P-LCR: large platelet rate.

Regarding the leukocyte series, the IG parameter was provided by six (37.5%) hematological analyzers; however, it was not released in the customer reports. Concerning the LIA index, 7 (70%) of the 10 laboratories whose equipment releases it provided this parameter in the customer reports. These data are shown in figure 2 , which represents the percentage of comparison between the parameters released by the hematological analyzers and those released in the customer’s report.

## DISCUSSION

In this study, CBC parameters released by hematological analyzers were compared with those released to customers in clinical analysis laboratories in a medium-sized municipality in the state of Minas Gerais, which is considered a regional health center in the Midwest region of the state. Our findings revealed that none of the clinical analysis laboratories released all parameters obtained in their hematological analyzers for the customer. This includes the most recent hematological parameters, such as reticulocyte indices and the RDW parameter, which has been known for a long time and is part of the list of parameters that must be present in a CBC report, according to the PNCQ.

Red blood cell distribution width is a simple CBC parameter that measures the size heterogeneity of circulating erythrocytes and has been provided by automated hematological analyzers since 1968. ^( 11 )^ Although RDW is traditionally used to investigate the etiology of anemia, several studies have shown that a high RDW is associated with a poor prognosis in the general population and in patients with coronary artery disease, metabolic syndrome, and heart failure. ^( 12 , 13 )^

Some hematological analyzers release RDW in two ways: RDW-CV and RDW-SD, which are calculated as coefficients of variation in relation to the average size of the erythrocytes and the average of their sizes, distributed up to 20% above the histogram of the automated erythrogram. Both mathematical and statistical data complement the MCV assessment because they are more precise and less subjective than microscopic observations of anisocytosis. Lower values represent a more homogeneous population, indicating extreme normality, whereas higher values correspond to the numerical expression of anisocytosis observed under a microscope. ^( 14 )^

Red blood cell distribution width-CV correlates with microcytic MCV, whereas RDW-SD is more closely related to macrocytic MCV. This was demonstrated in a study conducted by Monteiro ^( 14 )^ involving 305 patients who were separated into three distinct groups based on the results of MCV: macrocytic (n=28), normocytic (n=256), and microcytic (n=21) patients. The results showed that, in the microcytic group, most (59%) presented RDW-CV results above the upper limit of normality, with a significant positive correlation with the MCV. In the macrocytic group, 78.5% had RDW-SD values above the reference limits, indicating a significant relationship with increased MCV values.

The presence of nucleated erythrocyte count (NBRC) in the peripheral circulation is a warning sign, except in newborns, as it can indicate mortality. Nucleated erythrocyte counts are immature erythrocytes present in the bone marrow during erythropoiesis that are absent in the peripheral circulation. In full-term newborns, NRBCs disappear from circulation 7 days after birth. However, low-weight newborns who suffer from conditions such as neonatal asphyxia and ischemic encephalopathy experience an increase in NRBCs in the peripheral blood, which is a predictor of postnatal morbidity and mortality. ^( 6 , 15 )^ Additionally, this index correlates with the hemolytic disease in the fetus or newborn, which is caused by the destruction of erythrocytes in the newborn or fetus by maternal antibodies. ^( 16 )^ In adults, the presence of NRBCs in the peripheral blood indicates high erythropoietic activity that occurs in sickle cell anemia, thalassemia, myeloproliferative syndromes, severe infection, severe acute hemorrhage, and hypoxia and is considered a marker of poor prognosis in critically ill patients. ^( 6 , 17 )^

May et al. ^( 18 )^ examined 421 adult patients in intensive care and observed that the hospital mortality rate was 42% in patients with NRBCs in peripheral blood. Additionally, they found that the higher the value of this parameter and the more days it was reported in the CBC, the greater the risk of death. Macichová et al. ^( 19 )^ showed that high levels of NRBCs and IGs also predict the risk of death in patients who are in critical condition under the evaluation of organ failure, in which the mortality rate was 30.5% (103 of 338) in patients with polytrauma, sepsis, and acute abdominal conditions.

Regarding the erythrocyte series, the number of reticulocytes and their indices enable real-time evaluation of erythropoietic activity. ^( 20 )^ Although not considered an integral part of the complete CBC, the evaluation of reticulocytes can be crucial for assessing patient health.

Anemia is a prevalent clinical condition in several countries, and its diagnosis remains challenging owing to the numerous underlying causes; therefore, it is crucial to conduct an adequate investigation through detailed examinations such as CBC for accurate diagnosis and treatment. ^( 1 )^ In this context, RET and reticulocyte indices, such as IRF and RET-He, obtained from hematological analyzers since the 1980s, play an important role in differentiating between regenerative and hyporegenerative anemias, monitoring patients who underwent therapeutic procedures such as bone marrow transplantation or chemotherapy, and monitoring the early response to intravenous iron therapy, respectively. ^( 21 - 24 )^ The accurate diagnosis and prognosis of a disease helps prevent complications and prescription errors, such as unnecessary iron supplementation for patients who have anemia due to causes other than iron deficiency. ^( 1 )^

Regarding the leukocyte series, some parameters, such as the LIA and IG indices, may contribute to improving the quality of the results released to patients. The LIA index was first described in 1907 and elucidated in detail from 1923 onwards. It corresponds to the count of large lymphocytes that are characterized by abundant and basophilic cytoplasm and the presence of some granules. ^( 25 )^ It plays an important role in the immune response to various conditions such as viral infections, infectious mononucleosis, dengue and COVID-19, hypersensitivity to medications, and immunological and lymphoproliferative disorders. ^( 26 )^ Further, this parameter is essential in cases of dengue, as it is elevated in individuals with severe clinical manifestations, such as respiratory compromise, hypotension, and hemorrhagic symptoms. ^( 27 )^

Hematological analyzers accurately account for this index; however, they cannot describe the morphological variations in lymphocytes caused by the various immunological stimuli present in inflammatory and infectious diseases, mainly those of viral origin and in neoplasms resulting in various alterations. Therefore, the terminology for these lymphocytes varies, and it is recommended to use the term “reactive lymphocyte” to describe lymphocytes of benign etiology, and the term “anomalous lymphocyte” should be used when there is suspicion of malignancy or clonal etiology. ^( 28 )^

Another important parameter is IG, which is the sum of the number of immature granulocytic cells (metamyelocytes, myelocytes, and promyelocytes). These cells are released from the bone marrow into the systemic circulation during bacterial infections, sepsis, inflammatory state, steroid therapy, trauma, myeloproliferative diseases, cancer, pregnancy, and surgeries; they are considered important markers of serious diseases. ^( 29 )^ The relevance of this parameter has been discussed previously. ^( 29 )^ However, the description of the maturation stages of granulocytes in the leukocyte differential count can mitigate the impact of not releasing this index in the CBC report.

With regard to the platelet series, PVI is useful in the differential diagnosis of thrombocytopenia, as it allows the differentiation of thrombocytopenia due to increased platelet consumption (hyperdestructive), in which PVIs are high, from those caused by insufficiency or suppression (hypoproductive) of bone marrow, in which PVIs are normal or reduced. Immature platelet fraction is an index that represents the percentage of new platelets produced by the bone marrow. It can be used as an early indicator of platelet recovery after transplantation or chemotherapy and as a risk factor for cardiovascular diseases. ^( 7 , 30 , 31 )^

Platelet volume indices have been available to professionals since the early 70’s and can be used to estimate platelet function, which is essential for the differentiation of thrombocytopenia. ^( 32 )^ Negash et al. ^( 33 )^ examined 83 patients divided into two groups, one with hypoproductive thrombocytopenia and the other with hyperdestructive thrombocytopenia, and found that the PVIs were significantly higher in patients with hyperdestructive thrombocytopenia than in those with hypodestructive thrombocytopenia. Additionally, they revealed that MPV and P-LCR have better sensitivity and specificity in discriminating between the two types of thrombocytopenia and can help avoid or delay invasive bone marrow aspiration and prevent unwanted platelet transfusion. Pogorzelska et al., ^( 34 )^ in a systematic review, reported an increased risk of type 2 *diabetes mellitus* in patients with high MPV, which is positively correlated with the value of fasting glycemia and the high value of glycated hemoglobin. Furthermore, an increased MPV has been observed in patients with cardiovascular diseases, cancer, and acute surgical conditions. Platelet distribution width, in turn, correlates with cardiac events as a result of platelet activation, indicating that PDW can predict left ventricular failure in patients with acute coronary syndrome, with a sensitivity of 81% and a specificity of 39%.

However, IVPs are indices that are considerably affected by analytical interference, such as collection difficulties, prolonged tourniquet use, lack of adequate homogenization, inadequate anticoagulant/blood ratio, the time between collection and CBC analysis, and the possibility of pseudothrombocytopenia due to platelet aggregation in EDTA and platelet satellitism. ^( 35 )^ These pre-analytical and analytical issues, as well as the difficulty in interpreting PVIs, may justify not including these indices in the customer’s CBC report.

In summary, CBC is one of the most requested examinations worldwide and it is important for the diagnosis of several diseases. However, not all parameters are released in the customer’s report, even though they do not incur additional costs for the laboratory and do not often require additional reagents. It is assumed that not all parameters are released in the customer’s report owing to technical issues, such as pre-analytical and analytical interference, lack of reference intervals for some parameters, standardization of the information contained in the report (post-analytical phase), and lack of knowledge in both physicians and laboratory professionals.

Considering these issues and the absence of these parameters in the CBC reports, it is necessary for clinical analysis laboratories to standardize the minimum parameters that must be reported in the CBC and seek measures to minimize and address possible interferences, as they are important parameters that have great relevance in clinical practice. Therefore, it is necessary to increase awareness regarding the importance of these parameters among health professionals. This may be done through health education actions, aiming to present the characteristics of each index mentioned in this study and reinforce the meaning of the increased or decreased levels for each reported parameter. Thus, this approach can enrich CBC and the services offered by laboratories to improve patient care. Further, it will be possible to reduce the number of cases with incorrect diagnoses and/or treatments, resulting in early and reliable investigations, thereby increasing the quality and life expectancy of the population.

This study has certain limitations. The necessary information was obtained through a questionnaire, and there may have been misunderstandings on the part of those who answered it, the possibility of the interviewees being influenced, and their willingness to provide the necessary information. However, in order to address these limitations, information about the hematological analyzers was obtained from the electronic websites and manuals of the manufacturers, and the reported parameters were checked in the reports released by the laboratories.

This study’s strength is that, to our knowledge, there are no other studies on this topic. Therefore, this study was the first study to address this subject and contemplate this important laboratory test result.

## CONCLUSION

All the clinical analysis laboratories evaluated in this study had at least one complete blood count parameter that was not released in the customer’s report, even though it was released by the hematological analyzers used. Therefore, it is important that health education actions are aimed at raising the awareness of health professionals, including those working in clinical analysis laboratories, about the importance of these parameters, as well as the standardization of minimum parameters to be released in the complete blood count reports.

## Appendix 1

Questionnaire for collecting data from laboratories


