# Supplementary material for: Hematological parameters: is there a difference between those released by the hematological analyzer and to the customer?
Source: Einstein (Sao Paulo). 2023 Dec 1;21:eAO0501. doi: 10.31744/einstein_journal/2023AO0501 (PMC10730264; doi:10.31744/einstein_journal/2023AO0501)
Supplement: Supplementary file 1 [file 2317-6385-eins-21-eAO0501-suppl01.pdf]

Appendix 1. Questionnaire for collecting data from laboratories

Questionnaire N° ___

Laboratory name: ____

Question 1. Laboratory size:

0 ( ) Large

1 ( ) Medium

2 ( ) Small

Question 2. Institution:

0 ( ) Public

1 ( ) Private

Question 3. Type of service:

0 ( ) Outpatient

1 ( ) Hospital

Question 4. What device is used to perform the blood count analysis?

____

Question 5. What method is used to perform the blood count analysis?

1 ( ) Electrical impedance

2 ( ) Flow cytometry

3 ( ) Other. Whih?____

Question 6. What parameters are released by the equipment used? Write down which ones.

____

____

Question 7. What parameters are released to the customer? Write down which ones.

____

____

____

____

____

____

____

____
